# Our Hot Future Has Arrived—Are We Prepared?

**DOI:** 10.1029/2023GH000936

**Published:** 2023-09-20

**Authors:** Gabriel M. Filippelli

**Affiliations:** ^1^ Environmental Resilience Institute Indiana University Bloomington Bloomington IN USA

**Keywords:** climate change, heat, health

## Abstract

Climate change has significantly enhanced dangerous heat events. Many of our institutions are ill‐prepared to provide science‐informed and rapid interventions to confront this. The GeoHealth community is working to bring science, public health, and medical professionals closer together to grapple with the challenges posed by extreme heat.

## Key Points

- The impacts of climate change and extreme heat on human health are increasingly severe
- Institutions need to better protect public health in the face of a warming planet
- GeoHealth initiatives are bringing climate scientists, public health professionals, and medical experts closer together on extreme heat

## 1 Article

“The Heat Will Kill You First: Life and Death on a Scorched Planet,” a book released in summer 2023 (Goodell, 2023), couldn't have landed at a more frighteningly appropriate time. As I write this during a welcome break in the blazing weather that has dominated the U.S., and many other parts of the planet, this year, I am struck by the future. In this case, global and often regional temperatures that may become typical 10 or even 20 years out seem to have landed here in 2023. This particular year has been exceptionally hot due to a double shot of climate change plus El Niño inter‐annual variability, conditions that are projected to continue through 2024. Of course, the first ingredient, global climate change due to continued emissions of warming gasses by us, is the general backdrop for this tragic play that we are acting out. El Niño will subside, but our altered climate will continue, and become more altered so long as we net more carbon emissions into the atmosphere than are removed.

Once we hit that balance of net zero emissions, and only then, will global temperatures stabilize at whatever level that will be. But are we prepared for the global health impacts of that future temperature? We were grossly unprepared for the public health crisis presented by summer 2023—why should we expect better in the future? Well, I have many reasons for optimism on this front, and the increased interest in the topic of GeoHealth is playing a central role in that feeling of hope. But first, what are the public health challenges that our hot present has brought us?

Extreme heat kills more people than all other “natural disasters” combined, but we are sorely behind in preparing for and acting on the health impacts of heat events. Two examples from the U.S. are illustrative of this. Heat relief policies for our various institutions, such as schools, are not uniform, are often locally implemented, and are not always based on best practices. Last week my colleague's daughter suffered heat illness when her school ill‐advisedly allowed outdoor play time at 2 in the afternoon for the kids to “run off energy,” all while the Heat Index was hovering around 115°F. Kids are far more susceptible to heat illness due to their size and metabolism, and thus there should be some uniform, even nationally developed, policies around heat protection.

A second example is from high school and college athletics, where a combination of exertion and high heat index can cause medical crises. One best practice to protect athletes who appear overheated is to “cool first, transport second” (to a medical facility), but the local response is often the other way around. Additionally, the best cooling method for a dangerous heat situation is having a reservoir of chilled water on hand for immediate immersion of the ill athlete, something that is not always available on training fields and sporting events. Finally, we have many ways to measure body temperature, including oral, aural, and skin, but these give inconsistent results and can be substantially different from the actual body temperature, which is indicated by rectal thermometers. But there is an aversion to using rectal thermometers among many, and thus many cases of a “cool” oral temperature will hide a dangerous internal temperature.

It is important of course to consider the preparedness of the broad range of institutions that support society, including health care, public safety, agricultural and building sectors. Grappling with the challenges to emergency medical services and pressures on Emergency Department staffing need to include extreme heat planning (e.g., van Loenhout et al., 2018). Challenges to public safety posed by impacts of heat waves on enhancing crime rates (Harp & Karnauskas, 2018, 2020) also need consideration of staffing and training of personnel. Outdoor labor is particularly vulnerable to heat morbidity and mortality due to the nature of the work (i.e., long hours outside in the sun typically without cooling), requiring development and adherence to heat safety standards (Morrissey et al., 2021).

The interdisciplinary dialog that the area of GeoHealth has spawned promises to grapple with these issues, and bring the science of temperatures and climate much closer to the science of individual health, and the policies that protect public health. The mantra of “measure, prepare, and act” is itself multi‐disciplinary, with climate scientists informing the measurement and prediction of extreme heat, public health officials developing and implementing plans to prepare countries, states, and towns to be more climate resilient, and health and disaster response professionals acting to step in and treat people who have been impacted. More and more initiatives are expanding the dialog and broadening the tent to include these sectors. Some are at universities, such as the Climate and Health program at George Washington University, the “Beat the Heat” program of the Environmental Resilience Institute at Indiana University (disclaimer as I am the Executive Director there), the Climate and Health program at the University of Colorado School of Medicine, the European Centre for Environment and Human Health at the University of Exeter, and the Climate Change Research Centre at the University of Sydney. Others are associated with national level programs, such as the new joint U.S. CLIVAR/National Institutes of Health Working Group on Climate and Health and the European Climate and Health Observatory, among others. Additionally, the U.S. National Science Foundation, through its GeoHealth INTERN program, is enhancing training opportunities for geoscience graduate students to work in the area of public health to grapple with some of these issues.

These institutions and initiatives are rapidly ramping up our capacity to measure, prepare, and act, and have commitments to doing so not just for the developed world but also for developing nations and regions. The challenges in developing regions are myriad, including the lack of instrumentation, underdeveloped public health institutions, and often inadequate infrastructure and funding support available to implement the changes needed to confront climate change and heat. Thus, much more attention needs to be placed on the health equity challenges (e.g., Manware et al., 2022) that are confronting our climate present, let alone our climate future.

Summer 2023 was a clarion call, a siren blaring emergency, that we should all heed. Because in the case of climate, heat, and health, and to borrow a phrase from Bachman‐Turner Overdrive, “baby you ain't seen nothing yet.”
